# Genetic import and phenotype specific alleles associated with hyper-invasion in *Campylobacter jejuni*

**DOI:** 10.1186/s12864-015-2087-y

**Published:** 2015-10-24

**Authors:** Abiyad Baig, Alan McNally, Steven Dunn, Konrad H. Paszkiewicz, Jukka Corander, Georgina Manning

**Affiliations:** School of Science and Technology, Nottingham Trent University, Clifton Lane, Nottingham, NG11 8NS UK; Wellcome Trust Biomedical Informatics Hub, University of Exeter, Exeter, UK; Department of Mathematics and Statistics, University of Helsinki, Helsinki, Finland; Department of Veterinary Medicine, University of Cambridge, Cambridge, UK

## Abstract

**Background:**

*Campylobacter jejuni* is a major zoonotic pathogen, causing gastroenteritis in humans. Invasion is an important pathogenesis trait by which *C. jejuni* causes disease. Here we report the genomic analysis of 134 strains to identify traits unique to hyperinvasive isolates.

**Methods:**

A total of 134 C. jejuni genomes were used to create a phylogenetic tree to position the hyperinvasive strains. Comparative genomics lead to the identification of mosaic capsule regions. A pan genome approach led to the discovery of unique loci, or loci with unique alleles, to the hyperinvasive strains.

**Results:**

Phylogenetic analysis showed that the hyper-invasive phenotype is a generalist trait. Despite the fact that hyperinvasive strains are only distantly related based on the whole genome phylogeny, they all possess genes within the capsule region with high identity to capsule genes from *C. jejuni* subsp. *doylei* and *C. lari*. In addition there were genes unique to the hyper-invasive strains with identity to non-*C. jejuni* genes, as well as allelic variants of mainly pathogenesis related genes already known in the other *C. jejuni*. In particular, the sequence of flagella genes, *flgD*-*E* and *flgL* were highly conserved amongst the hyper-invasive strains and divergent from sequences in other *C. jejuni*. A novel cytolethal distending toxin (*cdt*) operon was also identified as present in all hyper-invasive strains in addition to the classic *cdt* operon present in other *C. jejuni*.

**Conclusions:**

Overall, the hyper-invasive phenotype is strongly linked to the presence of orthologous genes from other *Campylobacter* species in their genomes, notably within the capsule region, in addition to the observed association with unique allelic variants in flagellar genes and the secondary *cdt* operon which is unlikely under random sharing of accessory alleles in separate lineages.

**Electronic supplementary material:**

The online version of this article (doi:10.1186/s12864-015-2087-y) contains supplementary material, which is available to authorized users.

## Background

*Campylobacter jejuni* is the major cause of gastroenteritis in humans worldwide. It is a self-limiting disease with the symptoms varying from watery diarrhoea with no inflammation to mucous containing bloody diarrhoea [1]. A rare but life-threatening consequence of *C. jejuni* infection is the neurological disorder Guillain Barré syndrome [2]. *Campylobacter* infections are mainly transmitted through poultry and related products, but unpasteurised milk products, contaminated water and other food products have also been implicated as potential sources [1, 3, 4].

*C. jejuni* is believed to be an invasive pathogen and invasion of, or translocation across, the intestinal epithelium is thought to disrupt the gut lining and bring about an inflammatory response resulting in diarrhoea. The presence of blood and mucous in the stool in some patients provides evidence of the invasive ability of *C. jejuni* [5]. Several studies have used *in vitro* cultured cell lines of human and non-human origin to investigate *C. jejuni* interaction with host cells [6–9]. In addition, *in vivo* animal infection models including primates have been used to study the role of invasion and adhesion in *C. jejuni* pathogenesis [10–13]. It is apparent that *C. jejuni*’s ability to adhere to and invade epithelial cells is influenced by the infection model or the type of intestinal cell line used [5–7]. It is also strain dependent, often influenced by the severity of the clinical symptoms [14]. One previous study quantitatively classified the invasion potential of *C. jejuni* strains isolated from clinical, poultry and environmental sources as hyper, high or low using an *in vitro* invasion assay with the hyper-invasive group consisting of a greater proportion of clinical isolates compared to isolates from other sources [15]. Further analysis of one of these hyper-invasive strains, 01/51, led to the identification of a number of genes associated with the hyper-invasive phenotype [16], and in particular that of the lipooligosaccharide (LOS) biosynthesis gene, *cj1136* [17].

Surface polysaccharides including LOS and capsule have been implicated in adherence and invasion of epithelial cells and in an *in vitro* ferret model of infection [18–21]. *C. jejuni* was only reported as having a capsule once the very first genome sequence was completed in 2000, and it was reported to be the major determinant of the Penner serotyping scheme [18, 22]. The capsular polysaccharide (CPS) region in *C. jejuni* in general has a similar structure to that in other organisms and is composed of three regions: two conserved regions encoding the proteins involved with assembly and transport which flank the central variable region composed of the genes involved in polysaccharide biosynthesis. The CPS region varies in size from 15 to 34 kbp with the central variable region consisting of 11–34 ORFs [23]. Recently, mosaicism in the CPS locus was reported with the presence of CPS genes elsewhere on the genome of *C. jejuni* strain X that were thought to add to the antigenic variability of the CPS [24]. *C. jejuni* capsules are known to contain *O*-methyl phosphoramidate (MeOPN) modifications which in *C. jejuni* NCTC11168 are synthesised by the products of *cj1415c-1418c* [25]. These genes, along with *cj1419c* and *cj1420c* have homologs in 61 % of the published CPS loci available [23]. The MeOPN modification of the CPS was shown to have a role in virulence in the *Galleria mellonella* model [26] and more recently loss of the MeOPN modification was shown to increase invasion into Caco-2 cells and decrease resistance to killing by serum [27]. A number of genes associated with heptose biosynthesis (*hdd*C, *gmh*A, *hdd*A and *dmh*A) have also been reported as being conserved amongst the CPS region in *C. jejuni*.

In this study we have investigated the group of hyper-invasive *C. jejuni* isolates at the whole genome level. We reveal mosaicism in the CPS region and elsewhere in the genome with the apparent import of genes from *C. jejuni* subsp. *doylei*, *C. lari* and *C. coli* which may contribute to the hyper-invasive phenotype of these strains. We also show that hyper-invasive strains do not belong to a defined lineage but are phylogenetically distributed across the species. However, the hyper-invasive strains all share identical gene sequences, or alleles, in a number of loci including key flagella genes and a second *cdt* operon not reported before in *C. jejuni*, indicating that these loci are strongly associated with the hyper-invasive phenotype.

## Results

### Hyper-invasive strains are distributed across the *C. jejuni* phylogeny

**Fig. 1:**
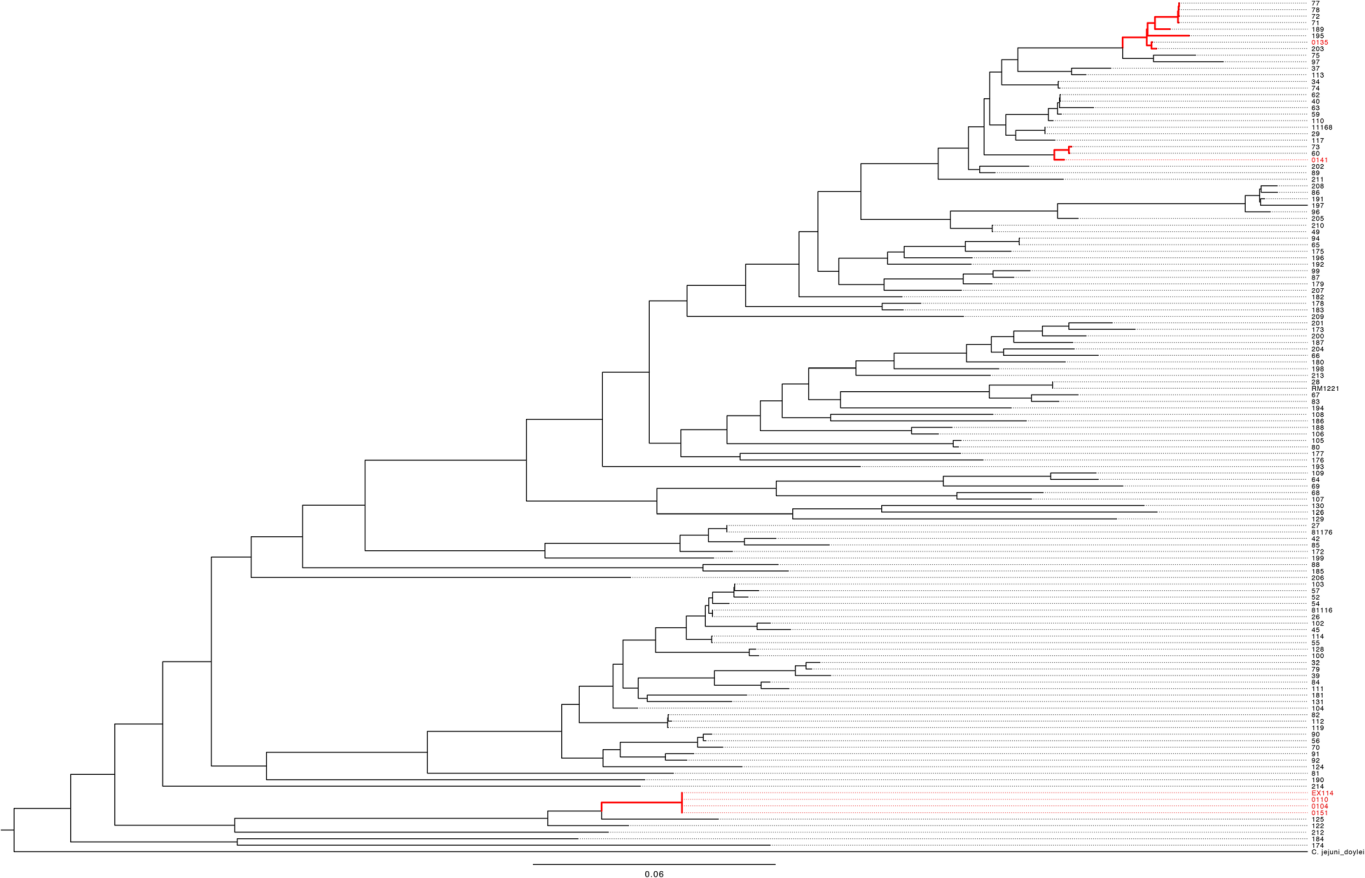
Maximum likelihood phylogeny derived from the core-genome alignment of 131 *C. jejuni* isolates. Isolates with a known hyper-invasive phenotype have their taxa identifier names highlighted in red. The three clades identified as containing hyper-invasive strains have branches indicated in red

All six isolates identified as hyper-invasive in our previous studies [15] (Table 1), were genome sequenced using the Illumina sequencing platform. The phylogenetic position of the six isolates was then determined from a core genome alignment incorporating all available *C. jejuni* reference genomes as well as the 128 genomes previously sequenced by Sheppard *et al*. [28]. The resulting phylogeny clearly showed that the hyper-invasive phenotype is not a lineage specific trait (Fig. 1). Three of the hyper-invasive strains (01/51, 01/35 and 01/41) are part of the host generalist and widely distributed clonal complex 21 [29], many of which have been assayed for invasion phenotype and shown not to be hyper-invasive [15]. These three strains are not grouped together and are randomly distributed across the phylogeny. Additionally, two further hyper-invasive strains, 01/04 and EX114, which belong to the clonal complex 677 and 682 respectively, are found at the opposite end of the species phylogeny, providing support for the hypothesis that hyper-invasion is an acquired trait that can occur in any *C. jejuni* lineage.

**Table 1:** List of strains used for pan-genome analysis in our study

Isolate	Background	Source	Accession number
01/51	Hyper-invasive clinical isolate	(Fearnley et al. 2008) [15]	ERS742291
			ERS742289
01/10			ERS742285
01/ 04			ERS742287
01/35			ERS742286
01/41			ERS742288
EX114			ERS742290
81116	Low invasive reference strain	(Fearnley et al. 2008) [15]	NC_009839.1
304	Low invasive Pig isolate		ERP006801
444			ERP006801
484			ERP006801
549_1			ERP006801
623			ERP006801
857			ERP006801

### Evidence of genetic import from *C. lari* and *C. doylei* in the capsular polysaccharide region of hyper-invasive strains

**Fig. 2:**
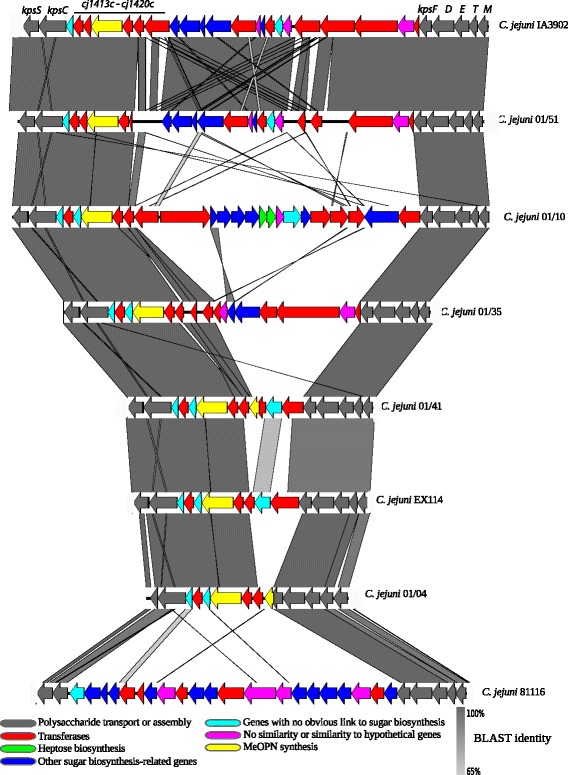
Comparison of the capsular polysaccharides (CPS) locus of the six hyper-invasive strains (*C. jejuni* 01/51, 01/10, 01/35, 01/41, EX114 and 01/41) with that in the low invasive strain *C. jejuni* 81116 and the hyper-virulent *C. jejuni* strain IA3902, isolated from aborted sheep. CPS loci from all strains were compared using BLASTn and visualised using EasyFig. CDSs are colour-coded to indicate putative gene function, with the conserved *kps* genes coloured grey. Grey scale indicates BLASTn similarity between CDSs

Since our earlier study identified a role for LPS modification in invasion [17], and a previous DNA microarray study (data not shown) indicated that the capsular polysaccharide region of *C. jejuni* strain 01/51 was divergent to that in other campylobacters, this region was studied in more detail in all six of the hyper-invasive genomes to investigate whether other surface structures might play a role in this phenotype. The overall structure of this region in the hyper-invasive strains is similar to other reported CPS loci with a central variable region flanked by conserved genes associated with capsule transport and assembly. The size of the locus varies amongst the hyper-invasive strains with strains 01/04 and 01/35 having the smallest locus of just 14, 746 bp and 01/10 having the largest locus of 35,655 bp, including the flanking transport and assembly regions. The number of genes in the central variable region ranges from just eight in strain 01/04 and 01/35 to 22 in strain 01/10. Genes associated with MeOPN modification (*cj1415c-1418c*) are present in all of the hyper-invasive strains, however the conserved heptose biosynthesis genes (*hdd*C, *gmh*A, *hdd*A and *dmh*A) are only present in one of these strains, 01/10. All of the hyper-invasive strains have a common set of six genes (*cj1413c*-*cj1420c*) adjacent to the *kps*C gene orthologue (Fig. 2), which are absent from the previously characterised low invasive strain 81116. This set of genes is also present in NCTC11168, which displayed high invasion levels in our previous study [15].

**Fig. 3:**
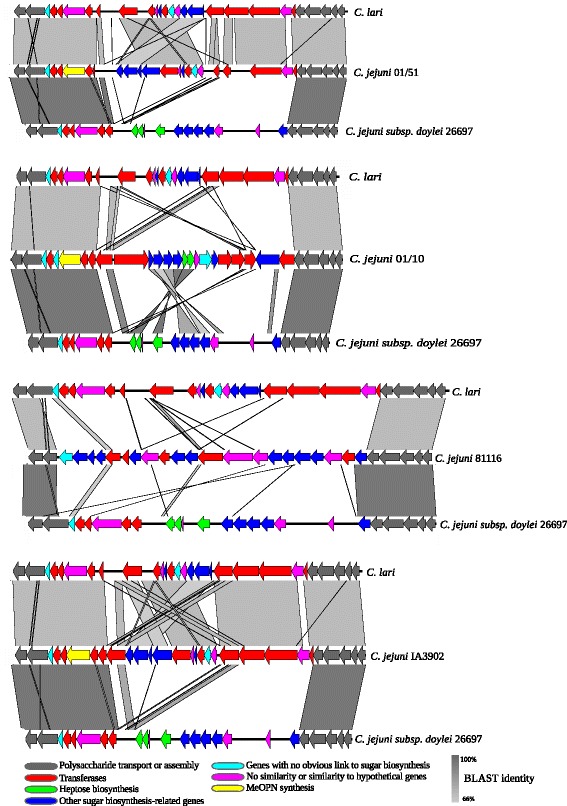
Comparison of the capsular polysaccharide (CPS) locus of two of the *C. jejuni* hyper-invasive strains (*C. jejuni* 01/51 and 01/10), *C. jejuni* 81116 and the hyper-virulent *C. jejuni* strain IA3902, isolated from aborted sheep, with the CPS locus from *C. jejuni* subsp. *doylei* 26697 and *C. lari* RM2100. CPS loci from all strains were compared using BLASTn and visualised in EasyFig. CDSs are colour-coded to indicate putative gene function. Grey scale indicates BLASTn similarity between CDSs

Comparison of the CPS locus across a wider set of *Campylobacter* genomes identified that the capsule locus for 01/51 was identical to that reported in the reference genome of *C. jejuni* strain IA3902, known to be a hyper-virulent strain, isolated from an aborted sheep [30] (see Fig. 2). BLASTn analysis of capsule genes with no identity to other known *C. jejuni* genes in both 01/51 and 01/10 returned high identity hits to CPS genes in *C. jejuni subsp. doylei* and *C. lari* (Fig. 3). This cross species similarity was also observed within the capsular variable region of IA3902, which as mentioned above was determined to be highly invasive (Fig. 3). This similarity was not observed for the capsule locus in *C. jejuni* 81116, which is the only available low invasive reference strain (Fig. 3). Our data suggests that the phylogenetically disparate hyper-invasive strains share similarities in import of non-*C. jejuni* genes into the capsule locus. We tested the ability of the hyper-invasive strains to survive exposure to human serum in comparison to low invasive reference strains and found no differences (data not shown).

### Core genome analysis revealed a number of hyper-invasive unique genes with similarity to genes from other *Campylobacter* species

Given our observation of genetic import at the capsule locus, and the lack of phylogenetic clustering of hyper-invasive strains, we decided to further investigate any other instances of shared gene content that may account for the observed hyper-invasive phenotype. A pan genome was constructed using LS-BSR, from the six hyper-invasive strains, *C. jejuni* 81116 and an additional six strains previously sequenced by our research group [31] that are known to be low-invasive. Group specific genetic loci were then identified using the accompanying compare-BSR.py script [32]. A number of loci were identified as either unique to the hyper-invasive strains or these strains had allelic variants of CDSs present in *C. jejuni* (Table 2). This finding was further confirmed by BLASTx comparison of each individual CDS to the entire non-redundant nucleotide database. This analysis verified that many of the hyper-invasion associated loci were either unique to the hyper-invasive strains or only had orthologues in other species such as *C. jejuni* subsp. *doylei*, *C. coli* and *C. lari* (Table 2), indicating possible import of these loci as observed in the capsule locus (Fig. 3). Many of these CDSs were involved in sugar and acyl transfer reactions or hypothetical proteins (Table 2).

**Table 2:** List of loci identified as associated with Hyper-invasive *C. jejuni* isolates

Locus^a^	Putative function^b^	BLAST identity results
Cj0151_00022	Hypothetical	Low identity across *Campylobacter* genus
*ruv*C	ribonuclease	Central portion unique
Cj0151_00042	acetyltransferase	No orthologs found
*flg*D	Flagella apparatus	Central portion unique
*flg*L	Flagella apparatus	N terminal unique
*flg*E	Flagellar hook protein	Central portion unique
Cj0151_00110	Hypothetical	No Orthologs found
hpaIIM	Methylase	No Orthologs found
Cj0151_00252	Hypothetical	Low identity across *Campylobacter* genus
Cj0151_00465	Hypothetical	Orthologs in *C. coli* and *Helicobacter spp.*
Cj0151_00778	Manosyltransferase	Low identity across *Campylobacter* genus
*fab*H_1	Oxo-acyl synthase	Single ortholog in *C. coli*
Cj0151_00944	Putative meticillin resistance	High identity across *Campylobacter* genus
Cj0151_00976	Hypothetical	Single ortholog in *C. coli*
*ugd*	Glucose dehydrogenase	No orthologs found
Cj0151_01062	Cj1136 LPS biosynthesis	Unique acetyl transferase domains
Cj0151_01198	Hemerythrin protein	Single ortholog in *C. coli*
*fha*A – *fha*B	Filamentous haemagglutinin	Low identity orthologs in *C. coli* and *C. fetus*
CdtA_2 – Cj0151_01598	Putative CDT operon	Orthologs to *cdt* operon of *C. lari* and *C. doylei*
CjEX114_01633	Nucleotide mutase	Single ortholog in *C. coli*
Cj0151_01634	Capsule sugar transferase	Low identity to *C. lari* ortholog

^a^CDS are annotated according to the genome annotation of our improved quality draft genome of isolate Cj0151

^b^Putative function is that ascribed to the CDS by Pfam and BLASTx searches

Interestingly, there was variability in sequence of some well-defined *C.jejuni* virulence genes with alleles conserved in all hyper-invasive strains. Most striking was a set of genes identified as a second *cdt* operon by BLASTx comparison. The *cdtA, B and C* genes showed 81 % similarity to gene BAJ52735 from *C. lari*, 81 % similarity to BAJ52756 from *C. lari,* and 83 % similarity to BAJ52757 from *C. lari* respectively. The secondary *cdt* operon was present in addition to the classic *cdt* operon found in abundance across the species. A visual comparison of the *cdt* loci identical to that performed for the capsule locus was undertaken. The nucleotide sequence similarity levels of 60–80 % suggest the secondary *cdt* operon is a paralog of the classical operon. However the secondary *cdt* operon showed more similarity at a phylogenetic level to the classical *C. jejuni cdt* operon than those of *C. jejuni* subsp. *doylei* and *C. lari*, and the orientation of the genes was in reverse to the classical *cdt* operon. (Additional file 1: Figure S1).

**Fig. 4:**
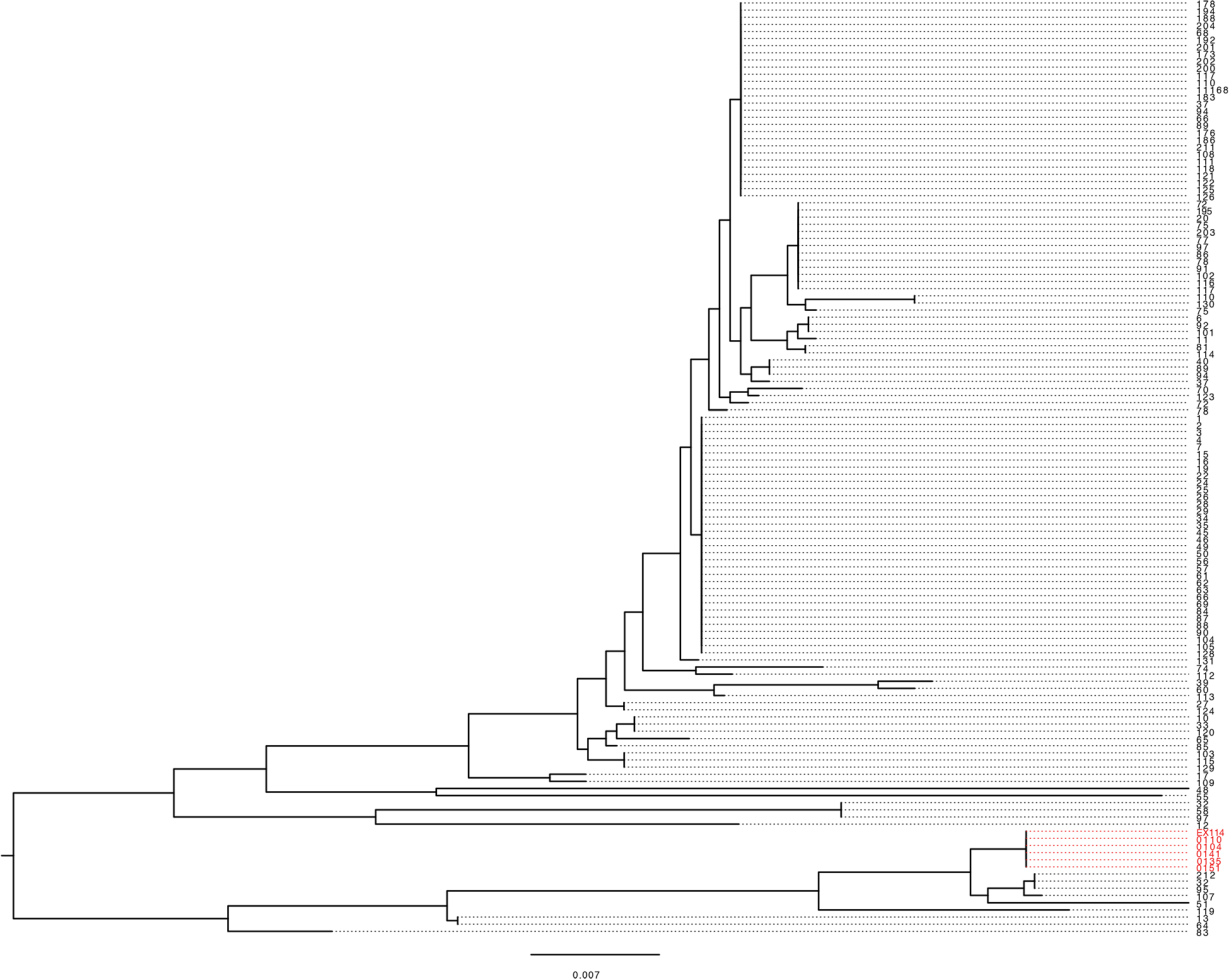
Maximum likelihood phylogeny of the concatenated sequences of *flgD, E* and *L* extracted from all 131 genomes used to create the core genome phylogeny. The hyper-invasive isolates are highlighted in red

**Fig. 5:**
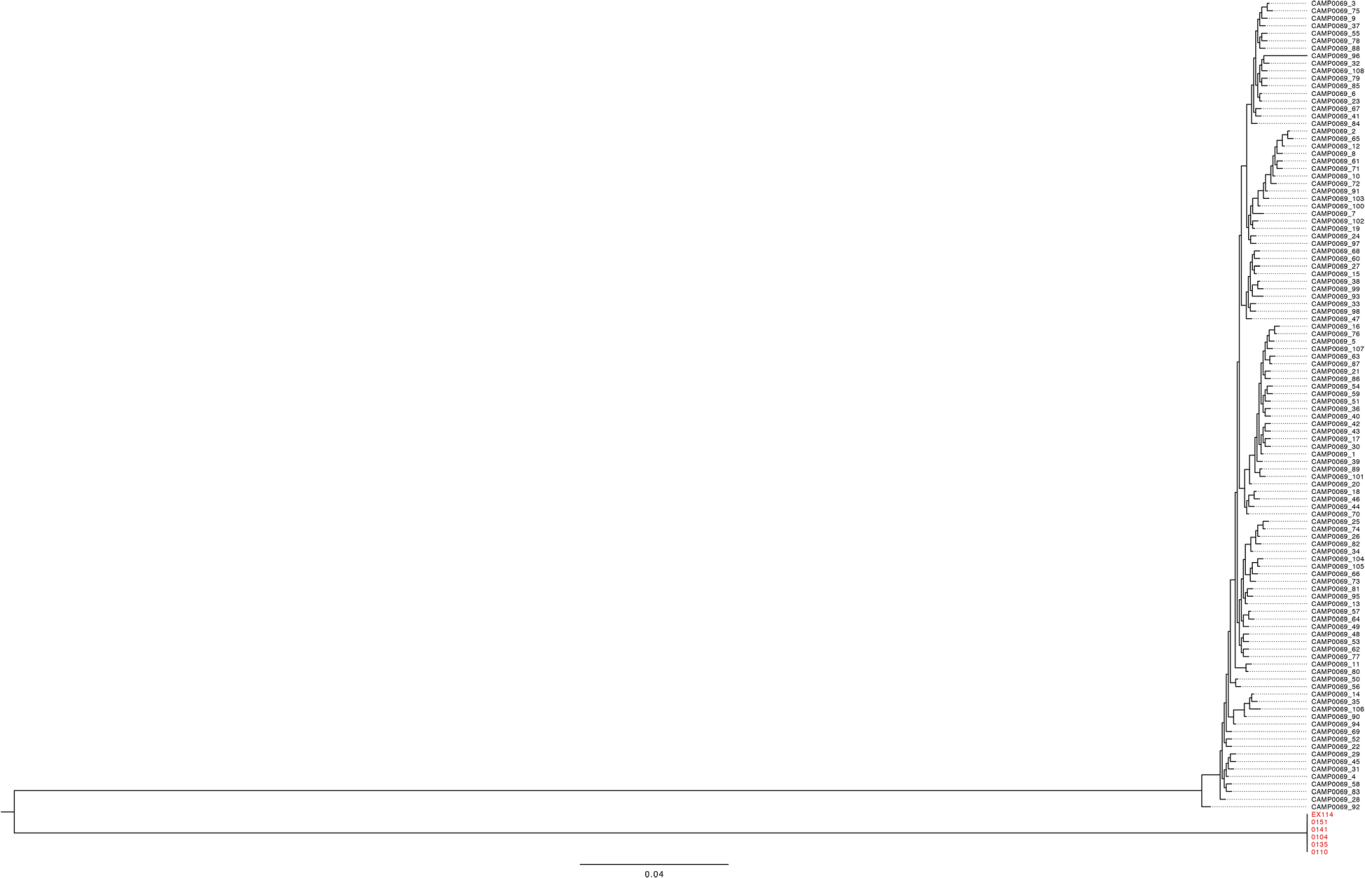
Maximum likelihood phylogeny of all available alleles of *cdtA* from the BigsDB Campylobacter database aligned with the *cdtA* gene identified in the secondary CDT operon of the hyper-invasive isolates. The hyper-invasive strains are highlighted in red

We focussed on the flagella genes and the secondary *cdt* operon and performed phylogenetic analysis of these regions. For the flagella genes, we extracted this locus from the core genome alignment and then determined a maximum likelihood phylogeny (Fig. 4). The phylogenetic tree showed comprehensively that *flgD, E* and *flgL* sequences are highly conserved in all six of the hyper-invasive strains and are divergent from the majority of versions found across the species. The hyper-invasive flagella genes form a distinct secondary clade that contains nine other *C. jejuni* strains that have not been characterised for invasion capacity (Fig. 4). For the second *cdt* operon, we aligned the sequences in hyper-invasive strains against all available *cdtA* alleles available on the *Campylobacter* BigsDB website (http://pubmlst.org/campylobacter/) and created a maximum likelihood phylogeny (Fig. 5). This approach was taken as the *cdt* operon was not present in every genome included in Fig. 1, and to ensure as comprehensive an analysis as possible against the full diversity of the *cdt* locus within *C. jejuni*. The phylogenetic separation of the *cdt* is even more apparent with the six hyper-invasive secondary *cdt* operons forming a highly divergent clade from all other *C. jejuni* alleles of *cdt* that have been sequenced (Fig. 5). BLAST analysis suggested the hyper-invasive *cdt* genes were similar to those found in *C. jejuni* subsp. *doylei* and *C. lari.*

To confirm that this allele sharing between the hyper-invasive strains was not a random event, we determined the likelihood of any given gene allele being shared between strains belonging to the three phylogenetic clades containing the hyper-invasive strains (clades marked in red in Fig. 1). A pan-genome matrix of all strains belonging to the three clades was created in LS-BSR and the number of alleles shared between these three clades measured. A total of 1551 genetic loci were compared and of those 53 shared an identical allele across the three clades containing hyper-invasive isolates, giving a probability of random allele sharing between these clades of 0.038. This provides strong statistical support that the *cdt* and flagella gene alleles shared between the hyper-invasive strains are not random and are associated with the shared phenotype of hyper-invasion.

## Discussion

Despite enormous research efforts and a significant body of literature examining the pathogenesis of *C. jejuni*, the virulence mechanisms of this highly diverse bacterial species remain somewhat enigmatic. A previous population phenotype study, uncovered very large diversity in the levels to which environmental and clinical isolates of *C. jejuni* invaded cultured intestinal epithelial cells *in vitro* [15]. The existence of isolates showing a hyper-invasive phenotype raised the possibility that a sub-population of *C. jejuni* exists which may cause a more invasive clinical disease [15]. Furthermore, the hyper-invasive strain 01/51 was previously studied by transposon and targeted mutagenesis and a number of genes involved in the hyper-invasive phenotype of this strain were identified [16].

In this study, we performed a comprehensive genomic analysis of all six isolates previously shown to display the highest levels of invasion an *in vitro* assay [15]. To place our results in the context of the wider *C. jejuni* population, we utilised the comprehensive genome sequence data produced by Sheppard *et al*. [28], investigating the ecology of *C. jejuni* lineages. The resulting whole genome phylogeny clearly showed the hyper-invasive phenotype is a trait that has been randomly acquired across distinct lineages. Three of our six hyper-invasive strains belong to the clonal complex 21, one of the most common host-generalist lineages of *C. jejuni* [33] which has been shown to contain low and hyper-invasive strains [15]. As such studies of genotypic and phenotypic traits associated with propensity to human pathogenesis have identified loci or traits that are phylogenetically distributed across the species [34].

Our previous research using DNA microarray showed that the hyper-invasive strain 01/51, lacked many capsule genes found in the other *C. jejuni* strains sequenced at that time (data not shown). A gene homologous to *cj1136* in *C. jejuni* NCTC11168, encoding for a glycosyltransferase with a role in the modification of LOS was shown to be essential for the hyper-invasive phenotype of *C. jejuni* 01/51 [17]. The capsule region in *C. jejuni* is postulated to play an important role in adhesion, invasion and increased virulence in surrogate infection models [26]. Owing to the importance of surface related structures in the pathogenesis of *C. jejuni*, we decided to investigate the capsule locus in detail in our hyper-invasive strains. Our data showed a high degree of variability in capsule locus architecture across the hyper-invasive isolates, perhaps not surprising given the phylogenetic distance between them, though interestingly the differences in architecture between the ST21-complex hyper-invasive isolates were just as great as they were across the more distantly related isolates. Despite this variability, there was a conservation of capsule locus genes with high similarity to genes found in *C. jejuni subsp. doylei*, *C. coli* and *C. lari*. Interspecies recombination has been shown to be a frequent event in the *Campylobacter* genus to the extent that it has blurred the species boundary in some lineages [35]. However, there are no reports in the literature of this being associated with a specific alteration in an important phenotype such as host-cell interaction. Wu et al. recently noted the similarity in the CPS locus of the hyper-virulent *C. jejuni* strain IA3902 and genes in *C. lari* and *C. jejuni* subsp. *doylei*, the latter frequently associated with bacteraemia [36, 37]. It has to be noted that there is limited data definitively linking these genes with the hyper-invasive phenotype with only one of our previous transposon mutants occurring in this locus [16]. However, the level of variability in the capsule locus suggests that the association between genotype and phenotype in this region is more complex than would be detected by classical single-gene targeted mutagenesis experiments. It may be that different combinations of genes can lead to an antigenically or structurally similar final capsule that confers the phenotype, but this can only be hypothesised when a robust study on the relationship between capsule genetics and final produced capsule is performed. In addition, the capsule locus of our 01/51 strains is completely identical to that found in the reference genome of *C. jejuni* IA3092. These two isolates are phylogenetically distant at the whole genome level, but *C. jejuni* IA3902 is classified as a hyper-virulent veterinary isolate of *C. jejuni* having been identified as the causative agent of highly aggressive infection in sheep resulting in abortive pregnancy [30]. Interestingly, despite the identical CPS locus in these two strains they differ in their Penner serotype with *C. jejuni* IA3902 having a serotype of HS:1,8 [36] and *C. jejuni* 01/51 with HS4 [17], confirming the fact that the CPS is not the sole determinant of the Penner serotyping scheme.

Further investigation into the pan-genome of the hyper-invasive *C. jejuni* isolates compared to a selection of low-invasive *C. jejuni* isolates, using the LS-BSR suite of tools identified a number of other loci associated with hyper-invasion. Such an approach has been successfully applied to identify genes associated with niche restriction in cattle-associated *C. jejuni* lineages that was also experimentally verified [38]. The genes found were involved with glycosylation of surface moieties and genes with hypothetical function. A LPS modification gene, *cj1136*, which was previously identified as being involved in the hyper-invasive phenotype of strain *C. jejuni* 01/51, by transposon and targeted mutagenesis was among the genes identified in our study, validating our analysis [17]. A large number of the loci also showed high identity with genes in *C. jejuni* subsp. *doylei*, *C. coli* and *C. lari* providing more weight to the suggestion that inter-species introgression of loci is strongly associated with the hyper-invasive phenotype.

Of most interest is our finding that the hyper-invasive strains carried specific alleles of flagella genes and the virulence associated cytolethal distending toxin genes. The phylogenetic analysis of these loci across the *C. jejuni* species provides strong evidence that these alleles are significantly associated with hyper-invasion. The flagella of *C. jejuni* have long been associated with virulence and invasion. Motility has been shown to be essential for cellular invasion [39] and flagella are also thought to act as the delivery machinery for the Type III secretion system Cia effector proteins which have been shown to facilitate invasion by *C. jejuni* [40, 41]. The *flgK, E,* and *L* genes identified in our screen all encode flagella basal body apparatus proteins suggesting that any form of antigenic variation in flagella in the hyper-invasive strains is unlikely. However, the structural or functional effects that these hyper-invasive specific alleles may have on flagella merits further detailed investigation. All previous transposon mutants in the flagella region of hyper-invasive strain 01/51 were discounted from further analysis due to the well-documented link between invasion and motility and re-characterising mutants in this region will allow us to further probe the link between hyper-invasion specific flagella behaviour and the invasive phenotype.

The most striking hyper-invasion associated allele is that of the secondary putative *cdt*, with all the hyper-invasive isolates containing *cdt* alleles which form a distinct secondary clade from all other *C. jejuni* alleles discovered to date. The cytolethal distending toxin has been suggested to be involved in modulating cytokine secretion by infected host cells and assist in intracellular survival of *C. jejuni* [42, 43]. However, clinical isolates have been discovered with natural deletion mutations in the *cdt* operon, and analysis of human sera suggests the CDT toxin is strongly immunogenic [44]. The secondary *cdt* allele associated with our hyper-invasive strains appears ancestral to the *C. jejuni* variants but is present in addition to the classic *cdt* operon found throughout the species suggesting it may be maintained in these strains for a defined purpose. BLASTx analysis of the entire non-redundant database identified just a single *C. jejuni* ortholog in strain 1336 [45] and no matching alleles for any loci in the *C. jejuni* BigsDB database. A detailed molecular analysis of these secondary *cdt* operon genes is now required to unravel their role in the hyper-invasion phenotype and their origin in these isolates.

## Conclusions

Here we present a comprehensive genomic analysis of a group of *C. jejuni* isolates previously shown by our group to exhibit a hyper-invasive phenotype *in vitro*. Our data suggests that genetic import from *Campylobacter* species other than *C. jejuni* is strongly associated with this phenotype. In particular, the highly variable capsule region displays patterns of genetic import from *C. jejuni.* subsp. *doylei* and *C. lari*. We also show that many loci identified as unique to the hyper-invasive isolates in a *C. jejuni* pan-genome analysis are orthologs of genes found in the other *Campylobacter* species. Finally, we show significant association between specific alleles of flagella and *cdt* genes and the hyper-invasive phenotype. Our data provides a targeted framework with which to further understand important phenotypic variations across the *C. jejuni* population.

### Data availability

The sequence data generated as part of this study can be found in the ENA (http://www.ebi.ac.uk/ena/data/view/PRJEB9504). The sequence data from the Sheppard et al. study can be found here: http://datadryad.org/resource/doi:10.5061/dryad.hk860?show=full.

## Methods

### Bacterial strains and growth conditions

A list of strains used in this study is provided in Table 1. All *Campylobacter* stocks were maintained at −80 °C as 1 ml aliquots in 20 % (v/v) glycerol in Muller Hinton (Oxoid) broth. The *Campylobacter* strains were routinely sub-cultured on mCCDA (modified *Campylobacter* blood free selective agar base, Oxoid) grown for 48 h at 37 °C in a microaerobic atmosphere (10 % CO_2_, 5 % O_2_, 85 % N_2_ v/v) in gas jars containing a CampyGen® pack (Oxoid) or in an anaerobic workstation (Don Whitley scientific, UK).

### Genome sequencing

The Qiagen genomic DNA extraction kit (Midi) (Qiagen, Crawley, UK) was used for the genomic DNA. Isolates 01/10 and 01/51 were sequenced on the Illumina Genome Analyzer IIx platform. Isolate 01/51 was dual sequenced, also on the Roche 454 platform. The 454 sequencing was performed to 12 x coverage of the *C. jejuni* 01/51 genome. The remaining isolates were sequenced on the Illumina HiSeq2500 platform by 150 bp Paired-end sequencing in a single multiplex run.

### Genome assembly & analysis

The combined data for isolate 01/51 was used to create an improved quality draft genome of a hyper-invasive isolate. The 454 sequence data was assembled using Newbler software, and the Illumina data was then mapped against the draft assembly using PAGIT [46]. For genomes sequenced on the Illumina platform, *de novo* assembly was performed using Velvet [47] and corrected using PAGIT [46]. The annotation of the reference strain *C. jejuni* RM1221 was transferred onto the un-annotated assembled sequences using the Rapid Annotation Transfer Tool (RATT) implemented in PAGIT. GLIMMER [48] prediction was used to identify ORFs that were unique to the sequenced genome. These ORFs were annotated by BLAST searching the NCBI BLASTx tools.

### Phylogenetic analysis

Core genome phylogeny was determined for the strains listed in Table 1, and the *C. jejuni* genome data set previously produced by Sheppard *et al*. [28]. The core genome alignment and maximum likelihood phylogeny was determined using the parsnp programme implemented in Harvest suite of tools [49], resulting in a core genome of 733,991 bp. The resulting phylogenetic tree was visualised in FigTree. Phylogenetic analysis of flagella genes was performed by extracting the nucleotide sequence of loci from the core-genome phylogeny and determining a maximum likelihood phylogeny in Fasttree [50]. Phylogenetic analysis of the *cdt* loci was performed by downloading the nucleotide sequences of all available *cdtA* alleles from the *Campylobacter* BigsDB site (http://pubmlst.org/campylobacter/) and aligning by Muscle in SeaView before determining a maximum likelihood phylogeny with Fasttree. All phylogenetic trees were visualised in FigTree.

### Comparison of CPS region

The nucleotide sequence of the capsule locus, spanning *kpsS* to *kpsM,* from all six hyper-invasive strains and the reference strains of *C. jejuni* NCTC11168, 81116, IA3902, *C. jejuni* subsp. *doyeli* 26697 and *C. lari* RM2100 were extracted from their genome sequences. These nucleotide sequences were used to perform BLASTn comparisons and visualised as multiple alignments using EasyFig [51]. The putative function of CDS within the capsule regions was determined by BLASTx against the NCBI non-redundant database.

### Pan-genome and identification of hyper-invasion associated loci

A pan-genome of the isolates listed in Table 1 was constructed using LS-BSR [32]. The genomes were then categorised into hyper-invasive and non-hyper-invasive and unique loci identified using the accompanying compareBSR.py script. All unique loci were identified by BLASTx analysis against the NCBI non-redundant database and CampyDB. To measure the level of allele sharing between lineages containing hyper-invasive strains, a pan-genome of all isolates belonging to the three clades containing hyper-invasive strains was constructed in LS-BSR. The number of alleles shared across the three identified clades was measured by identity score.

## Additional file

Additional file 1: Figure S1. Comparison of the *cdt* operons of *C. lari, C. jejuni* subsp. *Doylei*, *C. jejuni* 81116 and the hyperinvasive O151 strain. Loci from strains were compared using BLASTn and visualised in EasyFig. CDSs are colour-coded to indicate putative *cdtA* (blue), *cdtB* (red) and *cdtC* (orange). The blue shaded scale indicates BLASTn similarity between CDSs. The Phylogenetic tree shows a maximum likelihood phylogeny of a concatenated alignment of the *cdt* operon from the hyperinvasive strains, the reference genome strains 81116, 81176, 11168 and M1 and the reference *C. jejuni* subsp. *doylei* 26697 and *C. lari* RM2100 genomes. (PDF 121 kb)
