# Supplementary figures and images for: Genetic import and phenotype specific alleles associated with hyper-invasion in Campylobacter jejuni
Source: BMC Genomics. 2015 Oct 24;16:852. doi: 10.1186/s12864-015-2087-y (PMC4619573; doi:10.1186/s12864-015-2087-y)

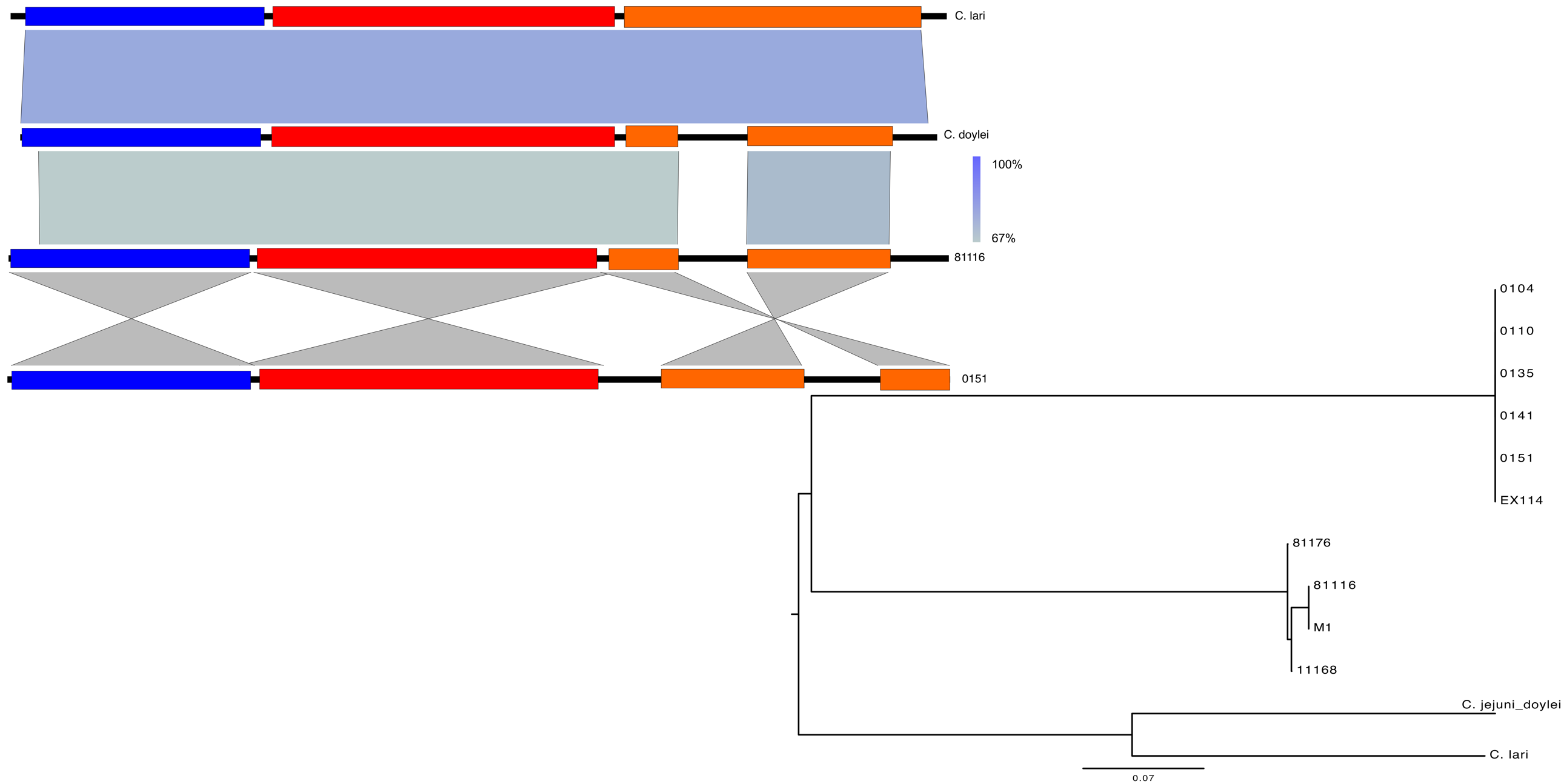

Supplement: Additional file 1: Figure S1. — Comparison of the cdt operons of C. lari, C. jejuni subsp. Doylei, C. jejuni 81116 and the hyperinvasive O151 strain. Loci from strains were compared using BLASTn and visualised in EasyFig. CDSs are colour-coded to indicate putative cdtA (blue), cdtB (red) and cdtC (orange). The blue shaded scale indicates BLASTn similarity between CDSs. The Phylogenetic tree shows a maximum likelihood phylogeny of a concatenated alignment of the cdt operon from the hyperinvasive strains, the reference genome strains 81116, 81176, 11168 and M1 and the reference C. jejuni subsp. doylei 26697 and C. lari RM2100 genomes. (PDF 121 kb) [file 12864_2015_2087_MOESM1_ESM.pdf]
